# Exploring the chicken embryo as a possible model for studying *Listeria monocytogenes* pathogenicity

**DOI:** 10.3389/fcimb.2014.00170

**Published:** 2014-12-10

**Authors:** Jonas Gripenland, Christopher Andersson, Jörgen Johansson

**Affiliations:** ^1^Department of Molecular Biology, Umeå UniversityUmeå, Sweden; ^2^Laboratory for Molecular Infection Medicine Sweden, Umeå UniversityUmeå, Sweden; ^3^Umeå Centre for Microbial Research, Umeå UniversityUmeå, Sweden

**Keywords:** *Listeria monocytogenes*, chicken embryo, PrfA, virulence: InlA, LLO, InlB

## Abstract

*Listeria monocytogenes* is a bacterial pathogen capable of causing severe infections in humans, often with fatal outcomes. Many different animal models exist to study *L. monocytogenes* pathogenicity, and we have investigated the chicken embryo as an infection model: What are the benefits and possible drawbacks? We have compared a defined wild-type strain with its isogenic strains lacking well-characterized virulence factors. Our results show that wild-type *L. monocytogenes*, already at a relatively low infection dose (~5 × 10^2^ cfu), caused death of the chicken embryo within 36 h, in contrast to strains lacking the main transcriptional activator of virulence, PrfA, or the cytolysin LLO. Surprisingly, strains lacking the major adhesins InlA and InlB caused similar mortality as the wild-type strain. In conclusion, our results suggest that the chicken embryo is a practical model to study *L. monocytogenes* infections, especially when analyzing alternative virulence pathways independent of the InlA and InlB adhesins. However, the route of infection might be different from a human infection. The chicken embryo model and other *Listeria* infection models are discussed.

## Introduction

*Listeria monocytogenes* is a Gram-positive human bacterial pathogen, capable of causing severe infections, Listeriosis, predominantly in immuno-compromised patients. Once ingested, the bacteria can cross the intestinal barrier and disseminate via the blood and lymph system to the liver and spleen, the primary sites for replication of *L. monocytogenes* (Vazquez-Boland et al., 2001; Lecuit, 2007). The route of infection follows a spatio-temporal pattern: In healthy hosts, the bacteria replicates for less than a week before being cleared by the immune system (Vazquez-Boland et al., 2001; Lecuit, 2007). In immuno-compromised patients, the bacterium continue to replicate and eventually disseminate into the blood-stream where it can cause septicemia. The bacteria may subsequently also pass the blood-brain barrier, leading to meningo-encephalitis or meningitis (reviewed in Vazquez-Boland et al., 2001; Lecuit, 2007). If the host is pregnant, the bacterium is capable of crossing the materno-fetal barrier resulting in abortion or severe neonatal infections. The latter phases of a *Listeria* infection have a mortality incidence between 20 and 30 % (Vazquez-Boland et al., 2001; Lecuit, 2007). To cross the intestinal barrier, *L. monocytogenes* uses an adhesin, InlA, which recognizes E-cadherin (Mengaud et al., 1996). Translocation through the placental barrier requires the concerted action of InlA and another adhesin, InlB (Disson et al., 2008), where the latter primarily recognizes the c-Met receptor (Shen et al., 2000). The molecular mechanism underlying blood-brain passage has not yet been resolved. Other proteins involved in cellular entry have also been recognized (i.e., Vip and Auto, Cabanes et al., 2004, 2005). Once internalized, *Listeria* is able to escape the phagosome through the action of Listeriolysin O (LLO), before it spreads from cell to cell by polymerizing actin in an ActA-dependent mechanism (Cossart, 2011). Almost all virulence factors are controlled by one transcriptional activator, PrfA, which bind PrfA consensus binding sites located in the promoter region of PrfA regulated genes, thus activating their expression (Freitag et al., 2009; de las Heras et al., 2011). Expression of PrfA is controlled at several different layers: At the transcriptional level through different promoters, at the post-transcriptional level by both a thermosensor lying in the 5′-untranslated RNA and by the action of small regulatory RNAs (Freitag et al., 2009; Gripenland et al., 2010). Finally, the activity of PrfA is believed to be controlled at the post-translational level, through a hitherto unknown PrfA binding factor (de las Heras et al., 2011).

## Animal model systems for studying listeria pathogenicity

Using cultured cell-lines, a tremendous knowledge of the different bacterial factors and cellular events that *Listeria* master has been gained. These cells are generally transformed cell-lines with different origins (cell-type and species), allowing a large choice for analyzing *Listeria* infection. To further understand the molecular mechanisms underlying *Listeria* infection, several animal models have been utilized. Accumulating *in vivo* animal data suggest that the mechanism of *Listeria* infection might be much more complex than previously anticipated, extrapolating from *in vitro* cell-culture data (Disson and Lecuit, 2013).

The mice (*Mus musculus*) model is frequently used to study a *Listeria* infection in a laboratory setting (Lecuit, 2007). The advantages are obvious; mice are relatively easy and inexpensive to maintain, the genome has been sequenced and a plethora of knock-out lineages are available for host studies. However, a drawback of using mice as a model for *Listeria* infections is the non-functional interaction between mouse E-cadherin and InlA, due to a glutamate instead of proline at position 16 of E-cadherin (Lecuit et al., 1999). This drawback has been resolved by replacing the glutamate to proline in position 16 of E-cadherin in mice (Lecuit et al., 2001; Disson et al., 2008) but also by “murinizing” InlA for it to recognize mice E-cadherin (Wollert et al., 2007).

Another model that has been used to study *Listeria* infectivity, albeit at a lower frequency, is the guinea pig (*Cavia porcellus*). Unlike mice, guinea pigs carry an E-cadherin recognizing InlA, which allows for an efficient translocation through the intestinal barrier (Lecuit et al., 2001; Khelef et al., 2006). However, the guinea pig was unable to provide a successful InlB:c-Met interaction, making this model system less suitable for studying the infection process downstream of intestinal crossing (Khelef et al., 2006).

Gerbil (*Meriones unguiculates*) which is a natural host *for Listeria monocytogenes*, allow InlA and InlB to successfully bind E-cadherin and c-Met, respectively (Disson et al., 2008). Therefore, the gerbil has been suggested as the animal of choice when studying *Listeria* infections. There are however drawbacks when using the gerbil model: The genome sequence of gerbils has not yet been published and they are not yet as commercially available as mice. Also, despite having functional InlA and InlB pathways, it was recently shown that very high doses of *Listeria* (~1 × 10^9^) were required to cause stillbirth in gerbils (Roulo et al., 2014).

The Rhesus monkey (*Macaca mulatta*), a non-human primate, has also been used to study *Listeria* pathogenicity. Its genome has been sequenced, the infection route is believed to closely mimic the human route and similar doses of *Listeria* cause stillbirth in both species (Smith et al., 2008). However, the Rhesus monkey model is expensive, requires large facilities and may be more ethically demanding.

Other animal infection models of *L. monocytogenes* include the zebra fish (*Danio rerio*), *Drosophila melanogaster* and *Caenorhabditis elegans* (Mansfield et al., 2003; Thomsen et al., 2006; Levraud et al., 2009). Their genomes have all been sequenced, they are inexpensive and optically accessible. Yet, since all three models are cold-blooded with a temperature maximum of 30°C, appropriate virulence factor expression might not reflect a human infection, which lies at 37°C.

Birds are natural hosts for *L. monocytogenes* and the bacterium has been associated with outbreaks in chicken broilers (Cooper et al., 1992; Vazquez-Boland et al., 2001). A possible avian model of *Listeria* infections is represented by chicken embryos (*Gallus gallus*). The chicken model has been used to assess the virulence of several pathogens, like: *Clostridium perfringens, Staphylococcus aureus, Escherichia coli, Salmonella enteridis*, and *Francisella tularensis* (Wang et al., 2008; Horzempa et al., 2010; Oh et al., 2012; Polakowska et al., 2012; Alnassan et al., 2013). Many studies have also investigated the pathogenic potential of clinically and environmentally isolated *L. monocytogenes* using chicken embryos (Terplan and Steinmeyer, 1989; Buncic and Avery, 1996; Norrung and Andersen, 2000; Olier et al., 2002, 2003; Severino et al., 2007; Yin et al., 2011). These reports clearly indicate that chicken embryos can be used as a model for *Listeria* infection, although the molecular mechanism causing the infection has not been examined in detail. The pore-forming cytolysin LLO have previously been shown to be important for chicken embryo infection (Jiang et al., 2005), but the impact of other PrfA-regulated factors have not been assessed.

## Using isogenic *L. monocytogenes* strains to infect chicken embryos

**Figure 1:**
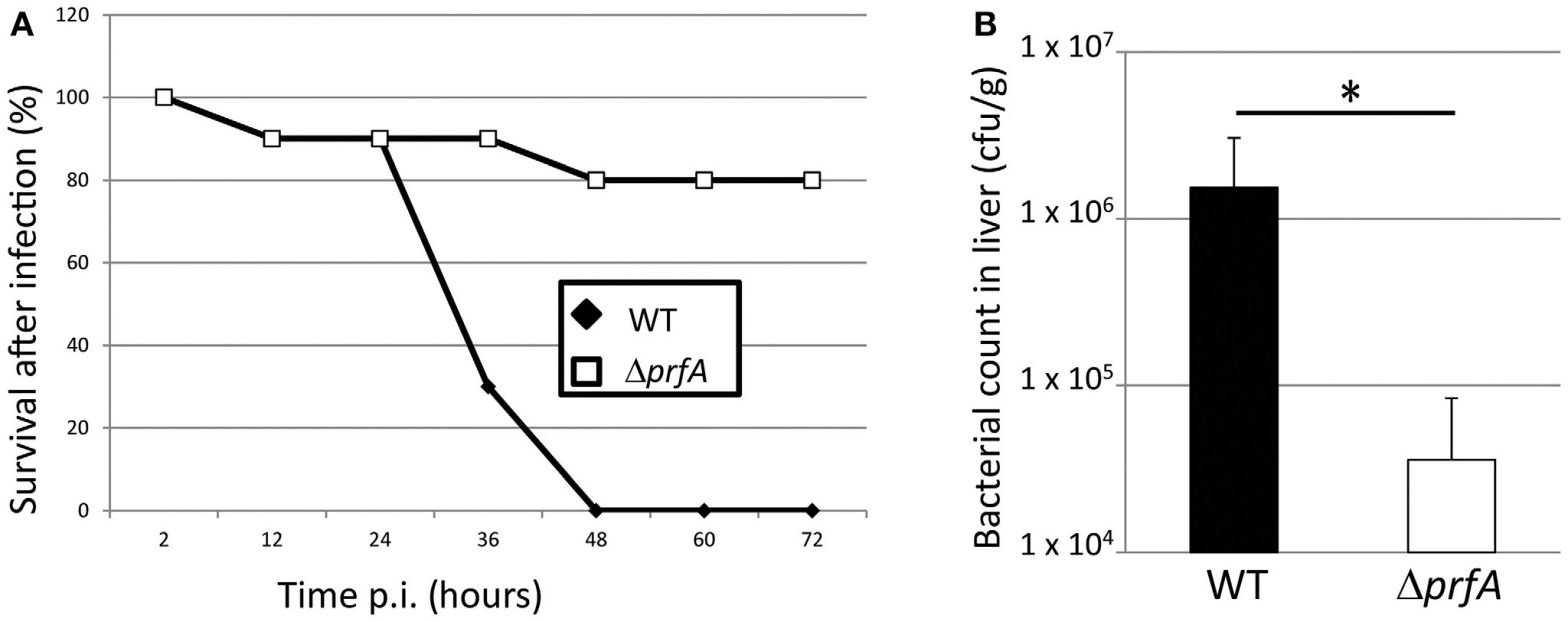
**(A)** Survival curve of chicken embryos infected with *L. monocytogenes* wild-type (EGDe) and the isogenic Δ*prfA* strain. ~5 × 10^2^ bacteria were inoculated into 9-day old chicken embryos, which were followed for 72 h by light candling. Death are shown as mean of 5 experiments (*n* = 17 for the WT strain and *n* = 16 for the Δ*prfA* strain). **(B)** Bacterial counts of wild-type and Δ*prfA* strains in the liver of chicken embryos. ~5 × 10^2^ bacteria were inoculated into 9-day old chicken embryos that were sacrificed after 34 h. The liver was isolated from living embyos and the number of viable bacteria was determined (*n* = 6 for the WT strain and *n* = 8 for the Δ*prfA* strain, divided over 3 experiments). Error bars show standard error. The difference is statistically significant (*p* < 0.05 through students *T*-test) and marked with an asterisk.

In order to examine the plausibility of using the chicken embryo model to assess *Listeria* pathogenicity as well as analyzing the importance of the PrfA virulence regulon, the EGDe (wild-type) strain and its isogenic Δ*prfA* strain were used (see Supplementary Materials for methods). Infecting eggs with 5 × 10^2^ wild-type bacteria lead to death of all eggs within 48 h (Figure 1A). Similar amounts of *Listeria* wild-type bacteria have been used in previous studies when infecting chicken embryos (Norrung and Andersen, 2000; Olier et al., 2002, 2003; Severino et al., 2007; Yin et al., 2011). Using the same amount of the Δ*prfA* strain, only 20 % of the chicken embryos were killed 72 h post-infection (Figure 1A). The inability of the Δ*prfA* strain to cause death of chicken embryos prompted us to investigate whether this was accompanied by a decreased infection (i.e. a reduced bacterial growth within the embryo). We therefore isolated livers from infected living embryos at 34 h post-infection. The wild-type infected embryos showed almost a 100-fold higher bacterial count compared with the Δ*prfA* strain in the liver (Figure 1B). We did not detect any obvious difference in the weight and appearance of the livers isolated from wild-type and Δ*prfA* strains (data not shown).

**Figure 2:**
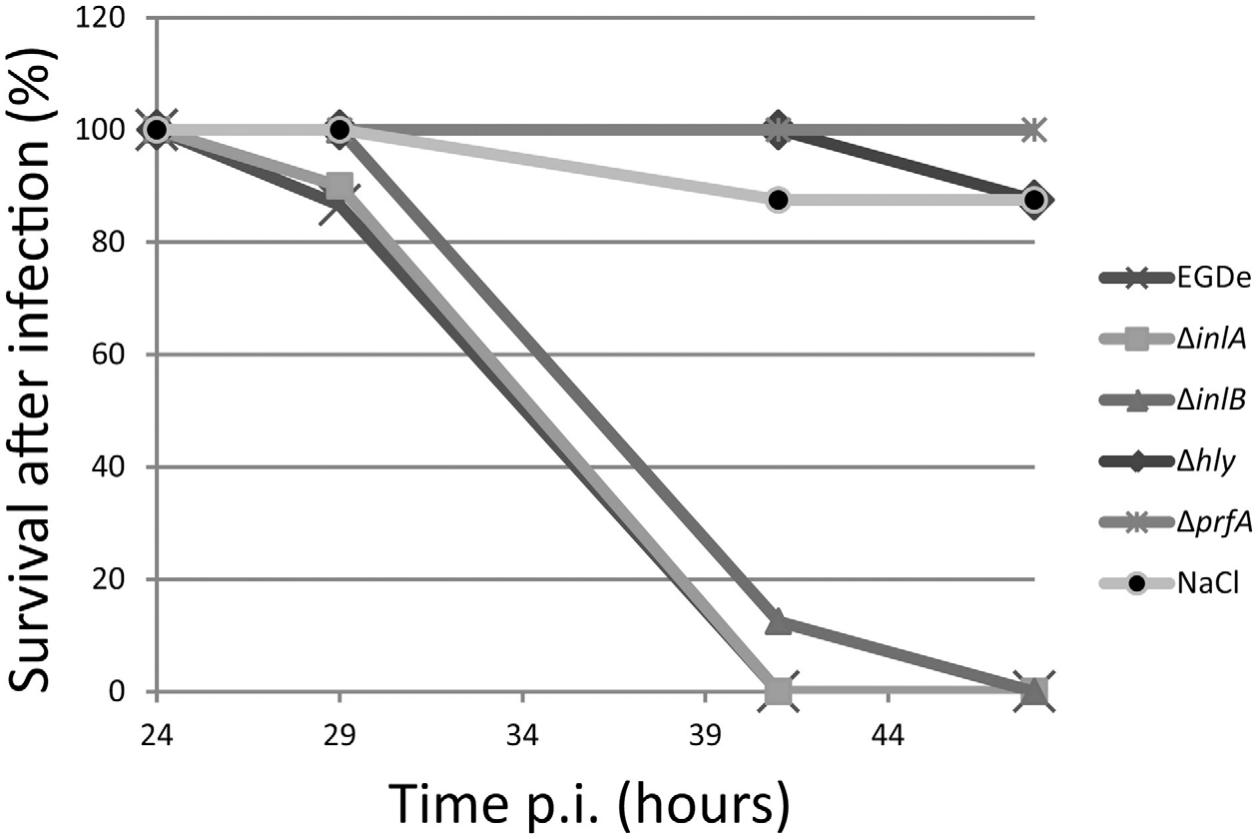
**Survival curve of chicken embryos infected with *L. monocytogenes* wild-type Δ*prfA*, Δ*hly*, Δ*inlA* or Δ*inlB* mutant strains**. ~5 × 10^2^ bacteria were injected into 9-day old chicken embryos and death was monitored from 24 to 48 h post-infection. Death is shown as mean of 2 experiments (*n* = 15, 9, 12, 11, 12, and 14 for WT, Δ*prfA*, Δ*hly*, Δ*inlA*, Δ*inlB*, and NaCl respectively).

To further examine the PrfA pathway and in more detail pin-point the role of certain PrfA-regulated virulence factors, isogenic mutant strains of *hly*; *inlA*, or *inlB* were used in a chicken embryo survival experiment together with the wild-type strain. The pore-forming cytolysin Listeriolysin O (LLO, encoded by *hly*) was required for a successful *Listeria* infection (Figure 2). This is in agreement with previous studies (McKay and Lu, 1991; Jiang et al., 2005), and highlights the importance of LLO during infection. *Listeria* infection of several epithelial cells require a successful adhesin: receptor interaction (Gaillard et al., 1991). The best characterized adhesins in *Listeria* are InlA, which recognizes E-cadherin, and InlB, which recognizes the c-Met receptor (Mengaud et al., 1996; Shen et al., 2000). Surprisingly, absence of either InlA or InlB did not attenuate *Listeria*-mediated killing of chicken embryos as compared with the wild-type (Figure 2).

## Discussion

In this work, we have analyzed if chicken embryos could be used as a model system for studying *Listeria monocytogenes* pathogenicity. Chicken embryos have previously been used as an infection model system to study the pathogenicity of various bacteria, including *L. monocytogenes* (Terplan and Steinmeyer, 1989; Buncic and Avery, 1996; Norrung and Andersen, 2000; Olier et al., 2002, 2003; Jiang et al., 2005; Severino et al., 2007; Wang et al., 2008; Horzempa et al., 2010; Yin et al., 2011; Oh et al., 2012; Polakowska et al., 2012; Alnassan et al., 2013). In this study, we have further assessed the chicken embryo as an infection model for *Listeria* using the well-used *L. monocytogenes* wild type strain EGDe. By examining isogenic mutant strains, we have also in more depth analyzed several virulence factors controlled by the transcriptional activator PrfA (i.e., LLO, InlA, and InlB). First, we established that the PrfA-pathway was essential to kill chicken embryos (Figure 1A). Also, almost a 100-fold lower number of Δ*prfA* bacteria compared with wild-type bacteria was observed between the strains when analyzing the liver (Figure 1B). When analyzing PrfA-regulated virulence factors, we observed that the pore-forming cytolysin LLO was absolutely required for a successful killing (Figure 2). The finding that both PrfA and LLO are essential for pathogenesis has been shown before (McKay and Lu, 1991; Vazquez-Boland et al., 2001). No one has however, to our knowledge, investigated the roles of the two most important internalins, InlA and InlB, in the chicken embryo model. In contrast to LLO, neither InlA nor InlB were required for a successful *Listeria* infection of chicken embryos (Figure 2). Similar to humans, the chicken has a proline at position 16 of E-cadherin, allowing a productive interaction between the adhesin InlA and its receptor E-cadherin (Lecuit et al., 1999). This is in contrast to mice which harbor a glutamic acid at position 16 of E-cadherin, preventing a successful interaction and hence invasion. A recent study could give a possible explanation of our results (Roy and Bandyopadhyay, 2014). In that work, the authors showed that the expression of the mRNA encoding E-cadherin was high early during the chicken development in all examined tissues, but diminished in chicken embryos older than 6 days. Since the chicken embryos in this study were 9 days or older, it could be hypothesized that the E-cadherin protein is less abundant and therefore play a less significant role during *Listeria* infection. The finding that InlB was not important for a chicken embryo infection is less surprising: The c-Met receptor in chicken embryos lacks lysines at position 599 and 600 in the c-Met receptor, which appear essential for a successful InlB:c-Met interaction in humans (Niemann et al., 2007).

Other groups have previously used chicken embryos to examine the virulence potential of clinically and environmentally isolated species of different serotypes by determining mean-time to death (Terplan and Steinmeyer, 1989; Buncic and Avery, 1996; Norrung and Andersen, 2000; Olier et al., 2002, 2003; Severino et al., 2007; Yin et al., 2011). We have instead focused this work on a defined bacterial lineage (EGDe). Nevertheless, many results obtained by other groups, using different *Listeria* lineages, overlap with our study. In particular, the survival curve of the embryo, after inoculation of the wild-type *L. monocytogenes* strain, was in agreement with previously published work (Buncic and Avery, 1996; Olier et al., 2002, 2003; Severino et al., 2007), indicating that the chicken embryo is a reliable model for studying *Listeria* infection. The chicken embryo model is well-established for studying development biology (Bénazéraf and Pourquié, 2013), as well as virus infections (Xia et al., 2013) where several tools have been developed. In light of this and through this work, we believe that the chicken embryo model might be a less-expensive but still reliable model for analyzing putative virulence factors compared to the mice model. However, since *L. monocytogenes* infection of chicken embryos is InlA and InlB independent, we believe that the chicken embryo model does not completely reflect the infection route in humans. It should be noted that *L. monocytogenes* can cause Listeriosis in avian species, like chickens (Cooper et al., 1992; Vazquez-Boland et al., 2001). The chicken embryo model would therefore be beneficial for the food-industry in order to examine route(s) of infection, but also means to prevent *Listeria* pathogenesis.

Although gerbils provide functional InlA and InlB pathways, the number of bacteria required to cause still-birth of pregnant gerbils is surprisingly high (Smith et al., 2008). The reason for this is unknown, but it could indicate that certain host factors that are required for an efficient *Listeria* infection are lacking in gerbils, thus making it a less attractive model. The situation for the chicken model is the opposite: *Listeria* strains lacking InlA or InlB still are able to kill chicken embryos.

In comparison with mice, the most studied animal model for *Listeria* infectivity, chickens also have a sequenced genome, are relatively inexpensive and easy to use. Listerial infection of chicken embryos also relies on LLO and PrfA for efficiency. Still, the mice model is preferable since an efficient infection require InlB (and InlA in the transgenic mice, Disson et al., 2008) indicating that this model more closely reflect human listeriosis. Also, the vast amount of mice knock-out mutants will promote the identification of host factors important for *Listeria* infection.

Despite having a c-Met receptor unable to interact with InlB, the guinea pigs still proves valid as a model organism to study *Listeria* infection pathways (Williams et al., 2009; Ebersbach et al., 2010; Wu and Matthews, 2013). In analogy to the guinea pig model, we speculate that *Listeria* uses alternative pathways for entering cells in the chicken embryo. We therefore suggest that the chicken embryo model could be used to identify novel *Listeria* virulence factors (e.g., other adhesins), that could be masked by an InlA/InlB-dependent mechanism in other animal models.

### Conflict of interest statement

The authors declare that the research was conducted in the absence of any commercial or financial relationships that could be construed as a potential conflict of interest.
