# Supplementary material for: Exploring the chicken embryo as a possible model for studying Listeria monocytogenes pathogenicity
Source: Front Cell Infect Microbiol. 2014 Dec 10;4:170. doi: 10.3389/fcimb.2014.00170 (PMC4261823; doi:10.3389/fcimb.2014.00170)
Supplement: Supplementary file 1 [file Supplementaryinformation.PDF]

Supplementary Materials, Gripenland *et al*

Materials and Methods

Bacterial strains and growth conditions. *Listeria monocytogenes* EGDe (1) and the isogenics strains: Δ*prfA*; Δ*hly*; Δ*inlA* and Δ*inlB* (2-4), were grown in BHI (Fluka) at 37 °C. Bacteria used for infection experiments were grown until OD_{600} = 1.0. Cells were harvested by centrifugation (6000 g, 10 min), and resuspended in 0.9 % NaCl, followed by appropriate dilution.

Infection of chicken embryos. Chicken embryos were housed in accordance with current Swedish guidelines. Chicken embryos (*Gallus gallus*) were incubated at 37.5 °C in a humidified rotary incubator (JANOEL) for indicated time-points, up to a maximum of 11 days. Eggs incubated for 9 days were sterilized with 70 % EtOH before 0.1 ml of bacterial culture (with concentration of bacteria or 0.9 % NaCl in negative control embryos) was injected with a syringe into the chorioallantoic sac of the embryo before re-incubation at 37.5 °C. The remaining hole was sealed with paraffin and adhesive tape. The infectious dose was determined at the time of infection by spreading suitable dilutions on LA agar plates (10). The eggs were examined 2 to 24 hours post-infection (depending on experiment) to identify lethal damages caused by the opening of the eggs and not associated with the bacterial infection *per se* and such eggs were removed.

Dead/live screening of infected chicken embryos. Dead/live screening of embryos was performed by *trans*-illuminating the eggs as shown previously (6-9, 11). Each egg was candled every 8-12h hour during the course of the experiments (72 hours). The following attributes was used to score the eggs viability: Presence and status of blood-vessels, embryo movement and fluid viscosity. All three parameters (Blood-vessel reduction abolished movement and altered viscosity) had to be fulfilled for us to conclude that death had occurred. Eggs were examined for viability during the entire course of the experiments.

Determination of liver colonization. Alive embryos were harvested at indicated time-points post infection, washed in 0.9% NaCl and isolated livers were homogenized (Dispomix) in 1 ml 0.9 % NaCl. Dilutions of samples were plated on BHI or LB agar plates which were incubated at 37 °C. The amount of colony forming units (CFU) per ml was determined and normalized by percentage to the amount of injected bacteria. The data is presented as a mean of the amount of CFU per gram of liver.

Statistical Methods. Error bars show standard error. * denotes statistically significant difference ($p < 0.05$) as determined by student t-test. Number of eggs (n) used is shown in the figure legends.

References

1. Glaser P, Frangeul L, Buchrieser C, Rusniok C, Amend A, Baquero F, et al. Comparative genomics of Listeria species. *Science* (2001) **294**(5543):849-52. Epub 2001/10/27. doi: 10.1126/science.1063447

294/5543/849 [pii]. PubMed PMID: 11679669.

2. Toledo-Arana A, Dussurget O, Nikitas G, Sesto N, Guet-Revillet H, Balestrino D, et al. The *Listeria* transcriptional landscape from saprophytism to virulence. *Nature* (2009) **459**(7249):950-6. Epub 2009/05/19. doi: nature08080 [pii]

10.1038/nature08080. PubMed PMID: 19448609.
3. Cabanes D, Sousa S, Cebria A, Lecuit M, Garcia-del Portillo F, Cossart P. Gp96 is a receptor for a novel Listeria monocytogenes virulence factor, Vip, a surface protein. *EMBO J* (2005) **24**(15):2827-38. Epub 2005/07/15. doi: 7600750 [pii]

10.1038/sj.emboj.7600750. PubMed PMID: 16015374; PubMed Central PMCID: PMC1182245.
4. Tiensuu T, Andersson C, Ryden P, Johansson J. Cycles of light and dark co-ordinate reversible colony differentiation in Listeria monocytogenes. *Mol Microbiol* (2013) **87**(4):909-24. Epub 2013/01/22. doi: 10.1111/mmi.12140. PubMed PMID: 23331346; PubMed Central PMCID: PMC3610012.
5. Terplan G, Steinmeyer S. Investigations on the pathogenicity of Listeria spp. by experimental infection of the chick embryo. *International journal of food microbiology* (1989) **8**(3):277-80. Epub 1989/06/01. PubMed PMID: 2642060.
6. Olier M, Pierre F, Lemaitre JP, Divies C, Rousset A, Guzzo J. Assessment of the pathogenic potential of two Listeria monocytogenes human faecal carriage isolates. *Microbiology (Reading, England)* (2002) **148**(Pt 6):1855-62. Epub 2002/06/11. PubMed PMID: 12055305.
7. Olier M, Pierre F, Rousseaux S, Lemaitre JP, Rousset A, Piveteau P, et al. Expression of truncated Internalin A is involved in impaired internalization of some Listeria monocytogenes isolates carried asymptomatically by humans. *Infection and immunity* (2003) **71**(3):1217-24. Epub 2003/02/22. PubMed PMID: 12595435; PubMed Central PMCID: PMCPmc148840.
8. Severino P, Dussurget O, Vencio RZ, Dumas E, Garrido P, Padilla G, et al. Comparative transcriptome analysis of Listeria monocytogenes strains of the two major lineages reveals differences in virulence, cell wall, and stress response. *Appl Environ Microbiol* (2007) **73**(19):6078-88. Epub 2007/08/21. doi: AEM.02730-06 [pii]

10.1128/AEM.02730-06. PubMed PMID: 17704270; PubMed Central PMCID: PMC2075013.
9. Yin Y, Tian D, Jiao H, Zhang C, Pan Z, Zhang X, et al. Pathogenicity and immunogenicity of a mutant strain of Listeria monocytogenes in the chicken infection model. *Clinical and vaccine immunology : CVI* (2011) **18**(3):500-5. Epub 2011/01/14. doi: 10.1128/cvi.00445-10. PubMed PMID: 21228136; PubMed Central PMCID: PMCPmc3067393.
10. Bertani G. Studies on lysogenesis. I. The mode of phage liberation by lysogenic Escherichia coli. *J Bacteriol* (1951) **62**(3):293-300. Epub 1951/09/01. PubMed PMID: 14888646; PubMed Central PMCID: PMC386127.
11. Norrung B, Andersen JK. Variations in virulence between different electrophoretic types of Listeria monocytogenes. *Letters in applied microbiology* (2000) **30**(3):228-32. Epub 2000/04/04. PubMed PMID: 10747256.
12. Ponce ML, Kleinmann HK. The chick chorioallantoic membrane as an in vivo angiogenesis model. *Curr Protoc Cell Biol* (2003) **Chapter 19**:Unit 19 5. Epub 2008/01/30. doi: 10.1002/0471143030.cb1905s18. PubMed PMID: 18228425.
